# A di-arginine ER retention signal regulates trafficking of HCN1 channels from the early secretory pathway to the plasma membrane

**DOI:** 10.1007/s00018-014-1705-1

**Published:** 2014-08-21

**Authors:** Yuan Pan, Joseph G. Laird, David M. Yamaguchi, Sheila A. Baker

**Affiliations:** Department of Biochemistry, Carver College of Medicine, University of Iowa, 51 Newton Road, Biochemistry, 4-712 BSB, Iowa City, IA 52242 USA

**Keywords:** Hyperpolarization gated channels, Photoreceptor, Neuron, Retina, Inner segment, HEK293, Targeting, Localization, Di-arginine, RxR

## Abstract

Hyperpolarization-activated cyclic nucleotide-gated 1 (HCN1) channels carry I_h_, which contributes to neuronal excitability and signal transmission in the nervous system. Controlling the trafficking of HCN1 is an important aspect of its regulation, yet the details of this process are poorly understood. Here, we investigated how the C-terminus of HCN1 regulates trafficking by testing for its ability to redirect the localization of a non-targeted reporter in transgenic *Xenopus laevis* photoreceptors. We found that HCN1 contains an ER localization signal and through a series of deletion constructs, identified the responsible di-arginine ER retention signal. This signal is located in the intrinsically disordered region of the C-terminus of HCN1. To test the function of the ER retention signal in intact channels, we expressed wild type and mutant HCN1 in HEK293 cells and found this signal negatively regulates surface expression of HCN1. In summary, we report a new mode of regulating HCN1 trafficking: through the use of a di-arginine ER retention signal that monitors processing of the channel in the early secretory pathway.

**Electronic supplementary material:**

The online version of this article (doi:10.1007/s00018-014-1705-1) contains supplementary material, which is available to authorized users.

## Introduction

Hyperpolarization-activated cyclic nucleotide-gated channels (HCN) are expressed in the brain and heart where they can modulate signal integration, synaptic transmission, or rhythmic changes in membrane excitability [1]. They are members of the voltage gated potassium channel superfamily and consist of four proteins, HCN1, HCN2, HCN3 and HCN4, that form heterotetramic or homotetrameric channels in a tissue-specific manner. The HCN channels are activated by hyperpolarization, and further modulated by cAMP. Upon activation, they carry inward currents consisting of K^+^ and Na^+^ [2]. Not surprisingly, disruption of their activity influences behavior and is pathological. For instance, alterations in HCN2 and HCN4 lead to cardiac arrhythmia [3]. HCN1 is more broadly expressed and influences behavior in diverse ways; it contributes to vision at medium and bright light intensities [4–6], memory formation [7], and sleep [8]. Perturbation of HCN1 underlies some forms of epilepsy and chronic pain [9–11]. The ongoing efforts to elucidate the mechanisms of HCN channel regulation are critical to making progress in understanding their roles in disease progression.

HCN channels are regulated by several convergent mechanisms. HCN channels open relatively slowly in response to membrane hyperpolarization and their gating is modulated by factors including cAMP, phosphatidylinositol 4,5-bisphosphate (PIP_2_), and the accessory subunit, tetratricopeptide repeat-containing Rab8b interacting protein (TRIP8b) [1]. Controlling subcellular trafficking is another important aspect of channel regulation but is not well understood. This is in part because there are likely overlapping mechanisms to account for the range of HCN localization found across cells.

HCN1 can be found broadly distributed throughout the plasma membrane of a neuron or be restricted to a particular compartment. In photoreceptors, only HCN1 is expressed; it is most abundant in inner segments and excluded from outer segments [5, 12–14]. In cochlear hair cells, HCN1 is localized to stereocilia [15, 16]. In hippocampal neurons, HCN1 is strikingly concentrated in distal dendrites [17]. In such neurons where HCN1 is concentrated in the distal dendrites, TRIP8b is the major regulator of HCN1 trafficking as it is essential for reducing axonal, but enhancing, dendritic localization of the channel [10, 18–21]. However, TRIP8b is not required to localize HCN1 to the plasma membrane of pre-synaptic terminals or retinal neurons [22, 23]. Determining how the localization of HCN channels in various cells and compartments is controlled is a current challenge for the field.

The cytoplasmic C-terminus of HCN1 and related channels is important for trafficking. Their C-termini contains two conserved structured regions, the C-linker which contributes to tetramerization and the cyclic nucleotide-binding domain (CNBD) which allows for modulation by cAMP, followed by a non-conserved region [24]. The non-conserved region contains the binding sites for TRIP8b [18, 20]. It also contains a binding site for filamin-A, which has been shown to promote clustering of the channel in the plasma membrane [25]. It is via the C-terminus that HCN1 and HCN2 bind KCNE2, a promiscuous potassium channel accessory subunit that increases the surface expression of its partners [26–29]. HCN3 surface expression is enhanced by its own accessory subunit, K^+^ channel tetramerization domain-containing protein 3 (KCTD3), which interacts with the portion of the C-terminus containing the C-linker and CNBD domains [30]. In a more distantly related Shaker-like K^+^ channel, Arabidopsis thaliana 2, specific residues in the C-linker are necessary for promoting surface expression of the channel [31]. It is not known how these factors influence HCN1 trafficking in different types of specialized cells such as photoreceptors.

Our purpose in this study was to identify additional regulatory elements controlling the trafficking of HCN1. We used *Xenopus laevis* photoreceptors, a powerful model system for dissecting neuronal and ciliary trafficking pathways [32]. Fusing the C-terminus of HCN1 to a reporter that alone accumulates in the modified primary cilia resulted in redirecting the reporter to the ER within the inner segment. By analyzing the localization of a series of mutants of this reporter-HCN1 CT construct, we uncovered a di-arginine ER retention motif. Di-arginine motifs have been found in a select subset of channels, and receptors and have been proposed to function as signals for improperly assembled channels. This predicts that disruption of the di-arginine motif in otherwise intact HCN1 channels would increase the amount of channel that exits the ER and localizes to the cell surface. Immunocytochemistry and biotinylation assays of HCN1 channels expressed in HEK293 cells verified that mutation of the di-arginine motif caused an increase in surface expression. In conclusion, a di-arginine ER retention signal that influences the trafficking of HCN1 from the ER to the plasma membrane has been discovered. We propose that this signal works in combination with as yet to be determined forward trafficking signals to ensure the proper delivery of functional HCN1 channels to the plasma membrane of neurons.

## Materials and methods

### Animals

*Xenopus laevis* were purchased from Nasco (Fort Atkinson, WI USA) and maintained by the Office of Animal Research at the University of Iowa. All experiments were approved by the Institutional Animal Care and Use Committee and adhered to the ARVO guidelines for animal use in vision research. Transgenic *Xenopus* tadpoles were generated using restriction enzyme-mediated integration as previously described [33, 34]). Briefly, linearized and purified plasmid DNA was integrated into sperm nuclei in the presence of a Ca^2+^-activated egg cytoplasmic extract. Treated nuclei were transplanted into unfertilized eggs obtained by inducing mature animals with human chorionic gonadotropin (Prospec, Ness-Ziona, Israel). Embryos were housed in 0.3× Marc’s Modified Ringer (30 mM NaCl, 0.6 mM KCL, 0.3 mM MgCl_2_, 0.6 mM CaCl_2_, 1.5 mM HEPES, pH 7.4). Transgenic tadpoles were identified at St 42 by screening for GFP expression in the eye and humanely euthanized between St 45 and 55 by immersion in 0.2 % tricaine (Sigma-Aldrich, St. Louis, MO, USA), prior to processing for immunohistochemistry.

### Molecular cloning

All constructs used for transgenesis were subcloned into the XOP5.5 vector containing the *Xenopus* opsin promoter to ensure rod photoreceptor-specific expression [35]. All inserts were generated by standard PCR protocols and verified by Sanger sequencing (Iowa Institute of Human Genetics, University of Iowa, Iowa, USA). The membrane reporter consists of the transmembrane domain from mouse activin receptor type 2A (aa 117–165), followed by EGFP, followed by a palmitoylated peptide from *Xenopus* rhodopsin (aa 311–349). The template for the *X. tropicalis* HCN1 inserts was obtained from retina cDNA; amino acid numbers thereby correspond to accession #XP002933077. The sequences of the primers used are provided in Supplemental Fig. 1. All constructs used for transfection of HEK293 cells were subcloned into the pEGFPN1 vector containing the CMV promoter. The mouse HCN1 insert was a generous gift from Dr. Yoav Noam [36, 37], the GFP-TRIP8b (1a-4) insert was a generous gift from Dr. Dane Chetkovich [36].

### Immunostaining

Transgenic tadpoles were fixed in 4 % paraformaldehyde (Electron Microscopy Sciences, Hatfield, PA, USA), cryoprotected in 30 % sucrose, and frozen in Tissue-Tek O.C.T (Electron Microscopy Sciences, Hatfield, PA, USA). Sections collected on charged glass slides were permeabilized in 0.5 % Triton X-100, blocked with 5 % goat serum and 0.5 % Triton X-100 in PBS, incubated in mouse α-GFP antibodies (diluted 1:500; Clontech Laboratories, Mountain View, CA, USA) or rabbit α-calnexin antibodies (diluted 1:100; Enzo Life Sciences, Farmingdale, NY USA), followed by secondary goat α-rabbit or goat α-mouse antibodies conjugated to Alexa 488 or 568 (Life Technologies, Grand Island, NY, USA) mixed with 2 μg/mL Hoechst 33342 (Life Technologies, Grand Island, NY, USA) to label the nuclei. Images were collected using a Zeiss 710 confocal microscope (Central Microscopy Research Facility, University of Iowa, IA, USA). Manipulation of images was limited to adjusting the brightness and contrast levels using Zen Light 2009 (Carl Zeiss Microscopy, Jena, Germany) or Photoshop (Adobe Systems Inc., San Jose, CA, USA). A minimum of four individual transgenic tadpoles were studied for every DNA construct.

### Cell culture

HEK293 cells (ATCC, Manassas, VA, USA) were maintained in DMEM supplemented with 10 % FBS, 250 μg/mL fungizone and 1 % penicillin/streptomycin (Life Technologies, Grand Island, NY, USA). For immunocytochemistry, cells were seeded at a density of 0.3 × 10^6^ cells/mL on chambered glass coverslips (Thermo Fisher Scientific Inc. Waltham, MA, USA,). A total of 0.4 μg plasmid DNA (0.2 μg for the HA-HCN1 constructs and 0.2 μg for the GFP or GFP-TRIP8b) was transfected using Lipofectamine 2000 (Life Technologies, Grand Island, NY, USA). 24 h post-transfection, cells were fixed in 4 % paraformaldehyde and stained as described above with α-HA antibodies (diluted 1:500; Thermo Scientific, Waltham, MA, USA). Each experiment was replicated a minimum of three times.

### Biotinylation assays

HEK293 cells were seeded at a density of 0.75 × 10^6^ cells/mL in 6-well plates and transfected with a total of 2 μg plasmid DNA (1 μg for the HA-HCN1 constructs and 1 μg for the GFP or GFP-TRIP8b) using Lipofectamine 2000 (Life Technologies, Grand Island, NY, USA). 24 h post-transfection, cells were incubated with 1 mg/mL sulfo-NHS-SS-biotin (Thermo Scientific, Waltham, MA, USA) for 15 min at 4 °C to label only the surface proteins. The reaction was quenched in TBS (50 mM Tris–Cl and 150 mM NaCl, pH 7.5) for 15 min at 4 °C, and washed in PBS. The biotinylated cells were homogenized in 200 µL lysis buffer (50 mM Tris–HCl, 10 mM NaCl, 0.32 M sucrose, 5 mM EDTA, 2.5 mM EGTA, 1.5 % Triton X-100, 0.75 % DOC and 0.1 % SDS, pH 7.4) supplemented with protease inhibitor cocktail (Complete, mini, Roche, Basel, Switzerland). Insoluble material was removed by centrifugation at 16,000×*g* for 10 min. 100 µL of the supernatant was collected and incubated with 50 µL NeutrAvidin agarose resin (Thermo Scientific Inc., Waltham, MA, USA,), with rotation at 4 °C overnight. Proteins bound to the beads were washed with PBS three times and eluted with 50 µL reducing NuPage LDS sample buffer (Life Technologies, Grand Island, NY, USA) followed by Western blotting with the following antibodies: rabbit α-HCN1 [23], rabbit α-TRIP8b [23], mouse α-NKA (M7-PB-E9, diluted 1:1,000; Santa Cruz Biotechnology, Dallas, TX, USA), rabbit α-GAPDH (diluted 1:500; Abcam, Cambridge, England), goat α-rabbit HRP and goat α-mouse HRP (Sigma-Aldrich, St. Louis, MO, USA). The software package Image Studio v3.1 (LI-COR Biosciences, Lincoln, NE, USA) was used for analysis of the blots. Each experiment was replicated a minimum of three times.

## Results

### The C-terminus of HCN1 redirects an integral membrane reporter to the ER in *Xenopus* photoreceptors

**Fig. 1:**
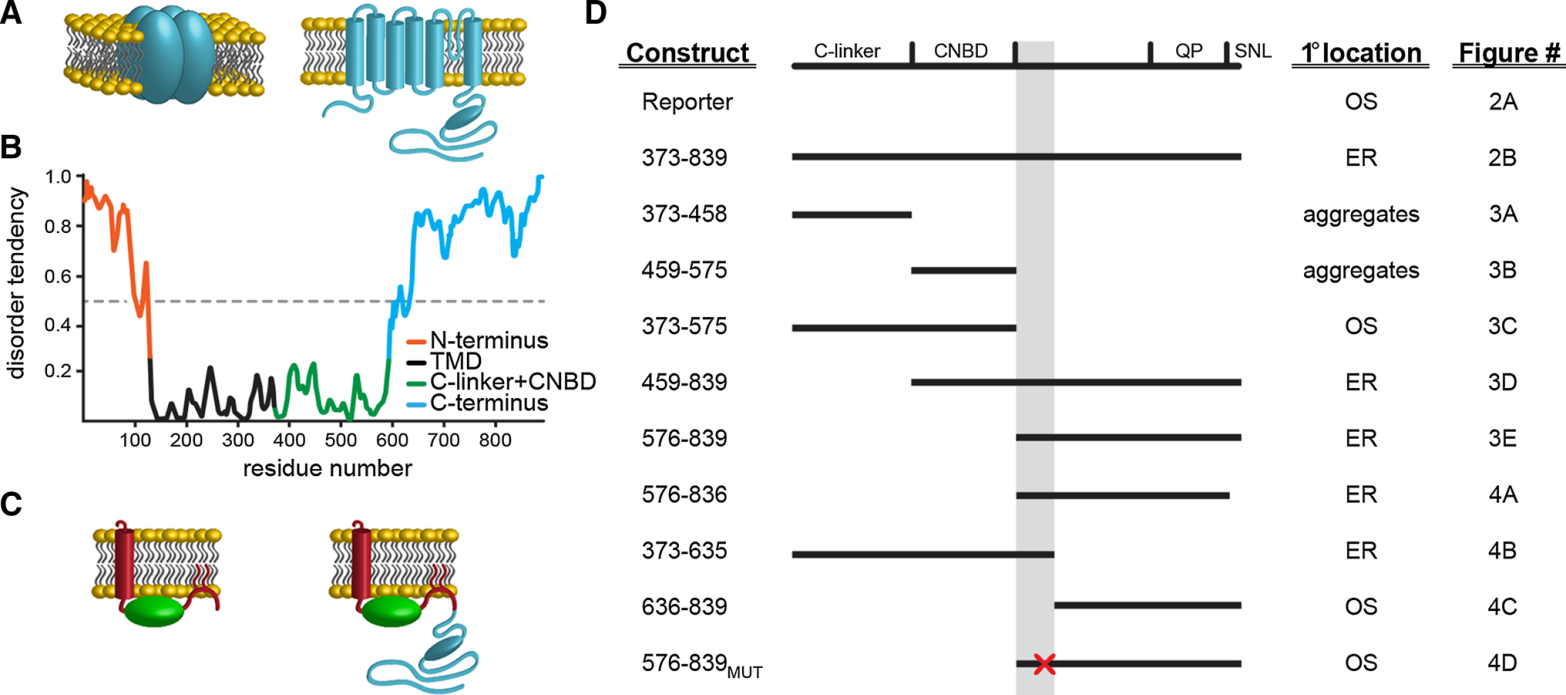
Overview of HCN1 structure and experimental design. **a** Schematic representation of an HCN1 homotetramer (*left*). Each monomer (*cyan*) consists of six transmembrane domains with cytoplasmic N- and C-termini (*right*). The *cyan oval* in the C-terminus indicates the relative position of the CNBD. **b** Disorder analysis of HCN1 using meta protein disorder prediction system. Residues above the threshold (*gray dashed line*) are predicted to be intrinsically disordered. **c** A cartoon of the reporter (*left*) consisting of a single pass transmembrane domain (*red*), GFP (*green*) and a palmitoylated peptide (*red*) to which the C-terminus (*cyan*) of *Xenopus* HCN1 (or portions thereof) was attached. **d** A summary of the design and targeting behavior of all constructs expressed in transgenic *Xenopus* photoreceptors. The linear arrangement of sequence motifs in the HCN1 C-terminus is displayed on *top*. The range of HCN1 amino acids attached to the reporter in each construct is listed in the first column. The *gray shaded area* is required for ER localization. *TMD* transmembrane domain, *CNBD* cyclic nucleotide-binding domain, *QP* glutamine- and proline-rich region, *OS* outer segment, *ER* endoplasmic reticulum

**Fig. 2:**
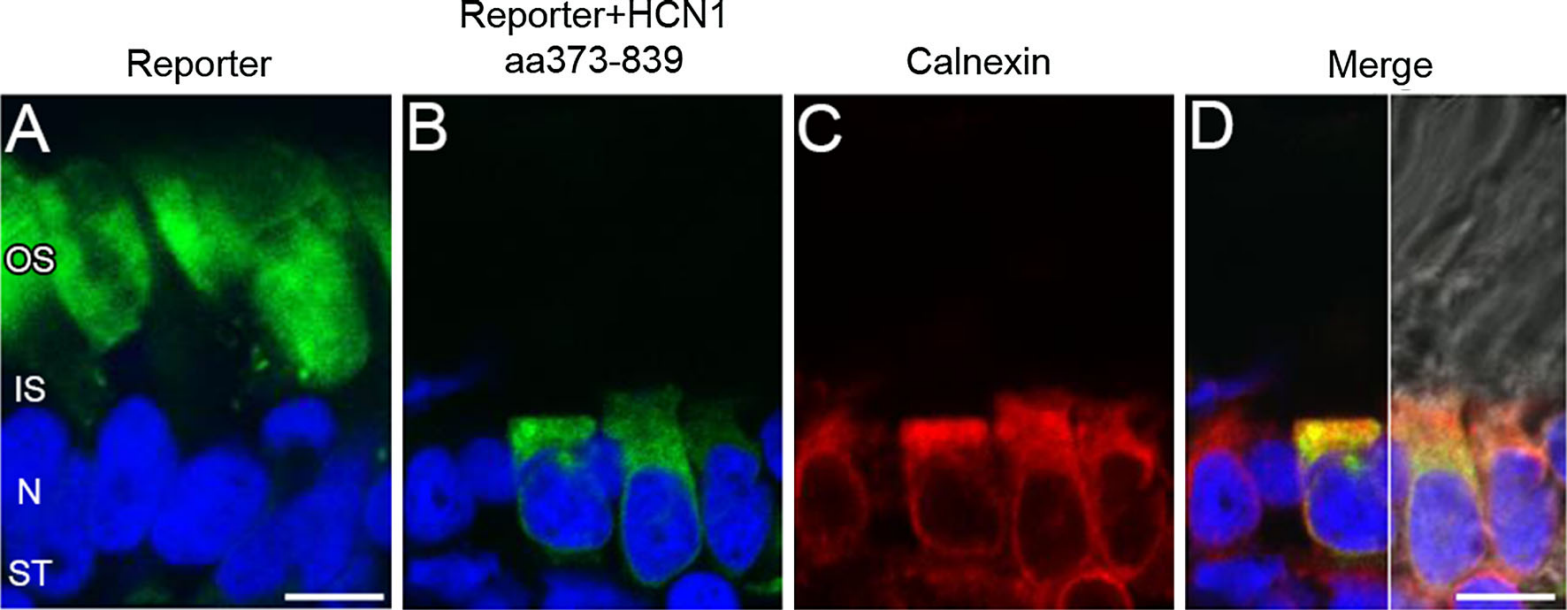
The C-terminus of HCN1 directs localization to the ER. **a** Transgenic *Xenopus* rod photoreceptors expressing the reporter alone or **b** in fusion to the cytoplasmic C-terminus (aa 373–839) of *Xenopus* HCN1. The transgenically expressed protein (**b**, *green*) colocalizes with calnexin (**c**, *red*) in the inner segment (**d**, transmitted light). *OS* outer segments, *IS* inner segments, *N* (*blue*) nuclei, *ST* synaptic terminals. *Scale bars* 5 μm

Photoreceptors express homotetrameric HCN1 channels. The major features of each monomer are an intrinsically disordered cytoplasmic N-terminus, followed by six transmembrane domains, and a cytoplasmic C-terminus. The membrane proximal portion of the C-terminus consists of the C-linker and cyclic nucleotide-binding domain (CNBD) followed by a long intrinsically disordered region (Fig. 1a–b) [1, 38]. To determine the contribution of the HCN1 C-terminus to trafficking of the channel in photoreceptors, we fused this sequence to a membrane reporter. This reporter consists of a single transmembrane domain anchoring GFP, which is followed by a peptide sequence that can be dually palmitoylated (Fig. 1c) [39]. The advantage of this reporter is that it accumulates in the outer segments when expressed in transgenic *Xenopus* photoreceptors (Fig. 2a), but relocalizes when fused to a protein sequence containing targeting information. Furthermore, as an integral membrane protein it will be co-translationally inserted into the ER, mimicking the initial steps of HCN1 trafficking. The membrane reporter fused to the C-terminus of *X.* *tropicalis* HCN1 (Figs. 1c–d, 2b) was found in internal compartments within the inner segment. This pattern is reminiscent of the distribution of ER in frog rod photoreceptors [40]. Immunostaining the same section with antibodies against calnexin (an ER resident protein) verified co-localization of the exogenous proteins with the ER (Fig. 2b–d). This suggested that the C-terminus of HCN1 contains an ER-targeting signal; a possibility tested with the series of deletion mutants described below and summarized in Fig. 1d.

### The ER-targeting signal is located within the intrinsically disordered portion of the C-terminus

We first tested if the C-linker or/and CNBD are responsible for the ER localization of the reporter-HCN1 CT construct. Note, the expression of fusion proteins containing the CNBD resulted in large numbers of intracellular aggregates and sometimes cell death in transgenic photoreceptors (data not shown). Incorporation of a single point mutation, R524E, previously shown to disrupt cyclic nucleotide binding but not the other functions of HCN1 [41], significantly reduced this problem. Therefore, the five constructs tested in *Xenopus* photoreceptors that contain the CNBD include the R524E modification. Using either the individual C-linker or CNBD domains fused to the reporter, resulted in proteins that were not localized to either the outer segment or ER.

**Fig. 3:**
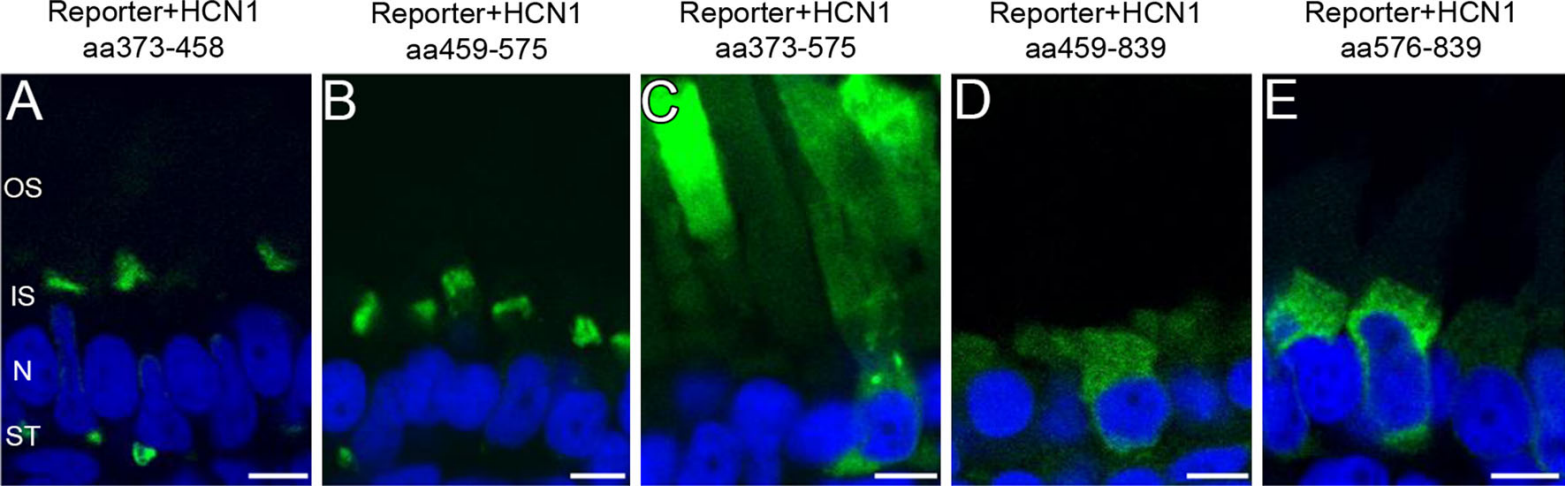
The C-linker and CNBD are not required for ER localization. Transgenic *Xenopus* rod photoreceptors expressing the indicated Reporter-HCN1 fragments detailed in Fig. 1d. *OS* outer segments, *IS* inner segments, *N* (*blue*) nuclei, *ST* synaptic terminals. *Scale bars* 5 μm

Instead, they were found accumulating in large amorphous structures in the apical portion of the inner segments and/or synaptic terminals (Fig. 3a–b). These structures may be extremely large aggregates or the isolation of these two structural domains may have exposed a cryptic association with mitochondria. Regardless, the problem was abrogated when the C-linker and CNBD were used in tandem. This caused the reporter to localize in outer segments (Fig. 3c). Appending the distal C-terminus to the CNBD or using just the distal C-terminus restored ER localization of the reporter (Fig. 3d–e). Together these data indicate that the ER-targeting signal is located downstream of the structured domains.

**Fig. 4:**
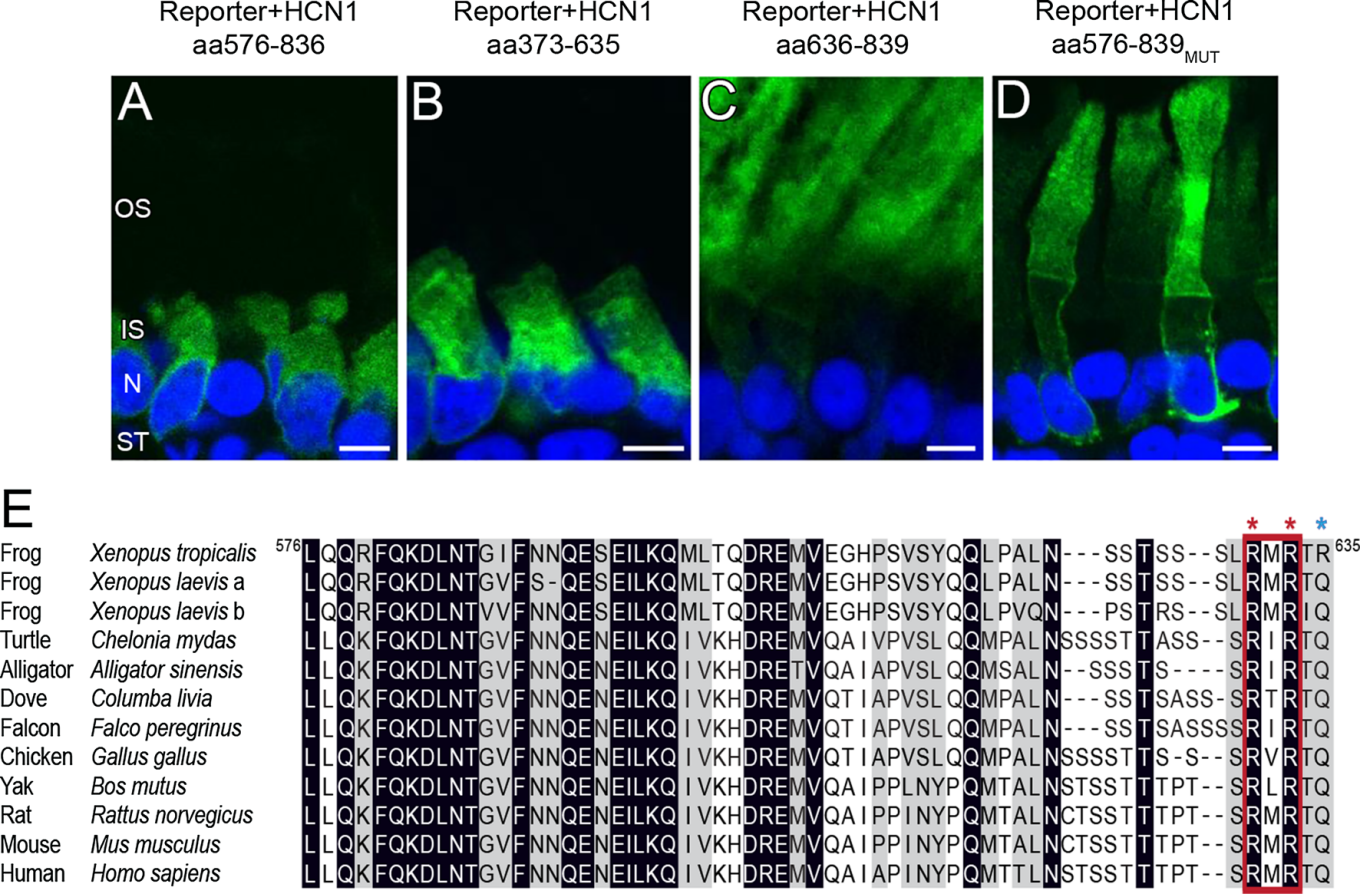
Identification of a di-arginine ER retention signal. **a**–**d** Transgenic *Xenopus* rod photoreceptors expressing the indicated Reporter-HCN1 fragments detailed in Fig. 1d. *OS* outer segments, *IS* inner segments, *N* (*blue*) nuclei, *ST* synaptic terminals. *Scale bars* 5 μm. **e** ClustalW sequence alignment of *X. tropicalis* HCN1 (aa 576–635) to HCN1 from various animal species. Identical residues shaded in *black*, partially conserved residues in *gray*, with the di-arginine motif outlined in *red* [*asterisks* mark arginines forming the RxR (*red*) or RxRxR (*blue*) motif]. Accession numbers are: *X. tropicalis*, XP002933077; *X. laevis* **a**, **b** deduced from genomic scaffold v7.1 52441 and 337825. *C. mydas*, XP_007052900; *A. sinensis*, XP_006017356; *C. livia*, XP_005500951; *F. peregrinus*, XP_005242028; *G. gallus*, XP429145; *B. mutus*, ELR46479; *R. norvegicus*, W9JKB0; *M. musculus*, O88704; *H. sapiens*, O60741

We next tested if any readily recognizable motifs in the distal C-terminus of HCN1 were responsible for the ER localization. Two regions drew our attention. There is a stretch of amino acids enriched in glutamines and prolines, that we call the QP region. The function of this region is currently unknown. Also, the last three amino acids (SNL) are highly conserved in HCN channels and serve as the binding site for the accessory subunit, TRIP8b [18, 20, 42]. Deleting the SNL signal did not prevent the ER localization (Fig. 4a), consistent with our previous finding that TRIP8b is not required for regulating HCN1 trafficking in photoreceptors [23]. Similarly, eliminating the QP region did not alter the ER localization pattern (Fig. 4b).

Comparison of all the constructs exhibiting ER localization tested thus far allowed us to constrain the region of interest to a shared 60 amino acid long region in the beginning of the disordered portion of the C-terminus (Fig. 1d). Deletion of this region resulted in localization of the reporter to outer segments (Fig. 4c, also Fig. 3c). Considering that motifs involved in protein targeting are often conserved across species, we examined an alignment of this region which drew our attention to a putative RxR di-arginine ER retention signal [43]. This motif is conserved among amphibians, reptiles, birds, and mammals (Fig. 4e). The sequence of HCN1 in *X. tropicalis* varies slightly from the other species in having two overlapping RxR motifs (RMRTR). Three point mutations (R631A, R633A, R635A) were made to test if either motif was functional. Indeed, this resulted in localization of the reporter to the outer segments (Fig. 4d, compare with Fig. 3e). In conclusion, a conserved di-arginine ER retention signal in the middle of the HCN1 C-terminus was responsible for the redistribution of the membrane reporter to the internal membranes of the inner segment.

### The HCN1 di-arginine ER retention signal regulates the amount of channel trafficked to the cell surface

Di-arginine motifs found in a number of channels and receptors are thought to mediate constitutive ER retention of partially folded or unassembled oligomers. Proper assembly of the protein complex likely causes a physical masking of the di-arginine motif, thereby allowing exit from the ER [44]. The robust ER localization we observed in *Xenopus* photoreceptors is consistent with this idea as the reporter constructs carrying only the C-terminus of HCN1 cannot assemble into a properly organized channel. To substantiate this, the requirement for the di-arginine motif should be tested in the context of the full-length HCN1. This is problematic in transgenic photoreceptors because the experimental versions of HCN1 would likely oligomerize with endogenous channels and override any mutations we incorporate. Therefore, we turned to a simpler model system for assessing the efficiency of ER exit for HCN1 channels.

**Fig. 5:**
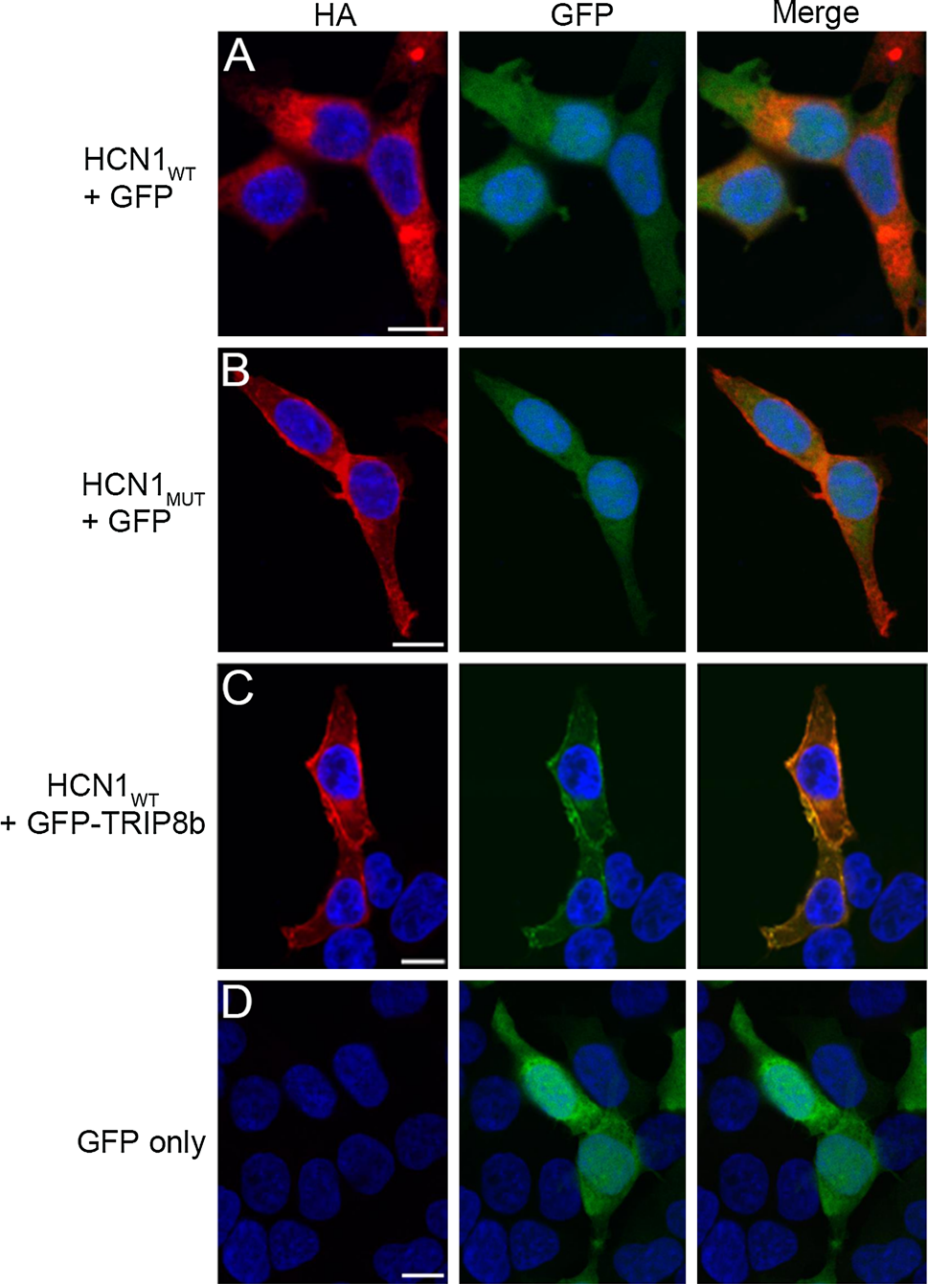
The di-arginine motif influences plasma membrane localization of HCN1. Immunostaining of HEK293 cells expressing the following proteins: **a** wild-type HA-tagged HCN1 and GFP; **b** HA-tagged HCN1 with a mutated ER retention signal (RxR to AxA) and GFP; **c** wild-type HA-HCN1 and GFP-TRIP8b; **d** GFP alone. HA tag (*red*), GFP (*green*), Nuclei (*blue*), and *scale bars* 10 μm

We expressed HA-tagged mouse HCN1_WT_ or HCN1_MUT_ (R648A and R650A, thus mutating the RxR motif to AxA) in HEK293 cells. Insertion of the HA tag in the second extracellular loop of mammalian HCN1 has been demonstrated to result in functional channels with normal biophysical properties when overexpressed in HEK293 cells [36, 37]. Immunostaining of transfected cells revealed that HCN1_WT_ was largely localized to the ER (Supplemental Fig. 2). However, plasma membrane localization of HCN1_MUT_ was prominent compared to that of the wild-type channel (Fig. 5a–b).

As a positive control, HCN1_WT_ was co-expressed with GFP tagged TRIP8b (1a-4), because this specific splice isoform of TRIP8b is known to increase surface expression of HCN1 [19, 21, 45]. Indeed the plasma membrane localization of HCN1_WT_ was dramatically increased (Fig. 5c). Note, in experiments lacking GFP-TRIP8b (1a-4), co-transfection of GFP was used to balance the amount of DNA transfected. In the negative control, only GFP was expressed to demonstrate specific HA immunostaining. The amount of plasma membrane HCN1 appeared greatest for HCN1_WT_ + TRIP8b, intermediate for HCN1_MUT_, and lowest for HCN1_WT_ alone.

**Fig. 6:**
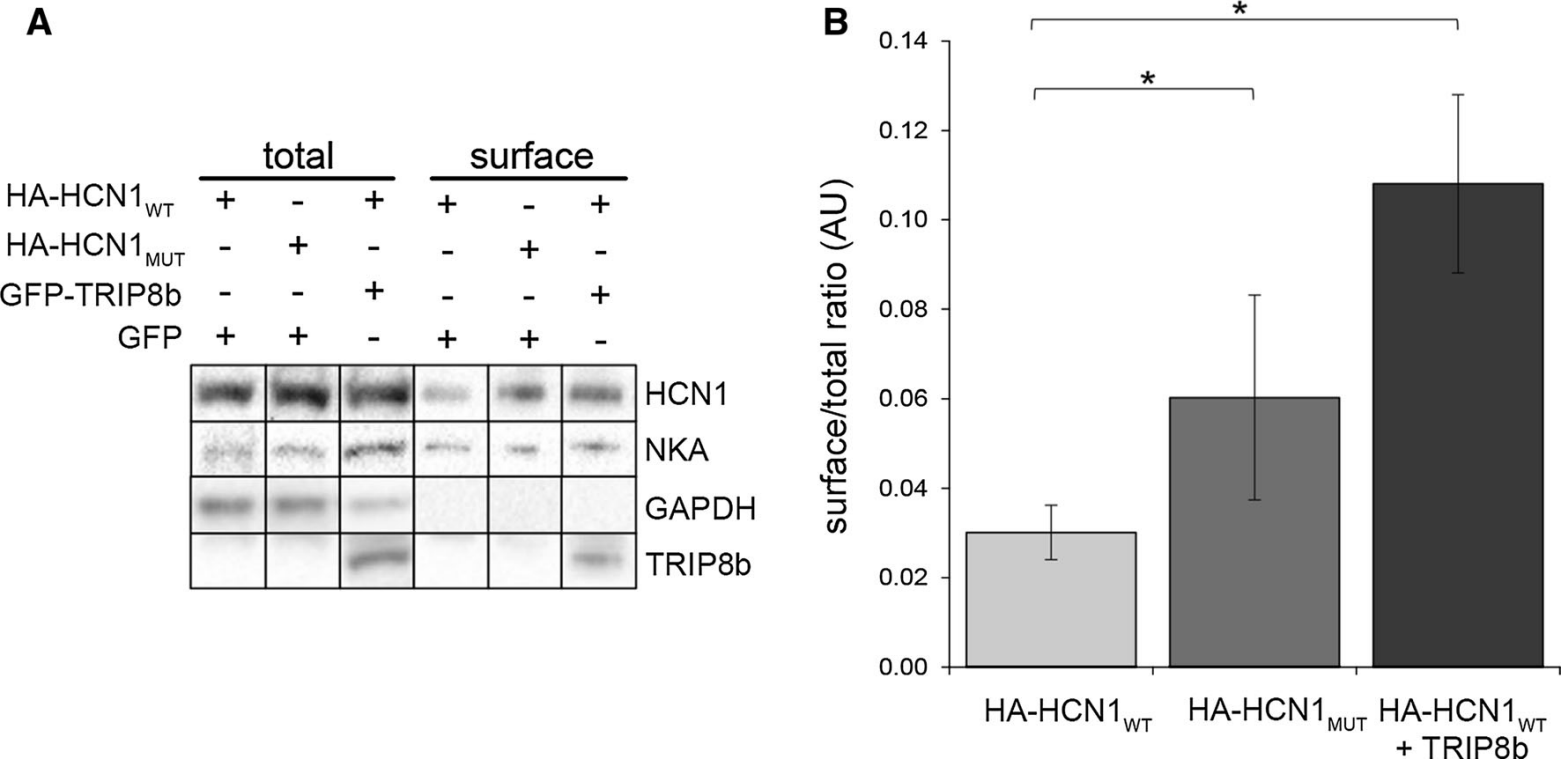
Mutating the di-arginine motif enhances the surface expression. Biotinylation assays of HEK293 cells transfected as described in (**a**, Fig. 5). **a** After biotinylation of transfected cells, surface proteins were pulled down using NeutrAvidin beads. The level of HCN1 and TRIP8b in the total and surface pools was detected by Western blotting. NKA and GAPDH were used as controls for membrane and cytosolic proteins, respectively. **b** Densitometry of Western blots represented in (**a**) was used to calculate the surface to total ratio of HCN1 after normalization to the loading control NKA. *Asterisks* indicate statistical significance with *p* < 0.01 (*t* test)

To quantify the surface expression of HCN1 we used biotinylation assays. Briefly, all proteins presented on the cell surface were labeled with a membrane-impermeable biotin reagent. The biotinylated surface proteins were subsequently isolated by an affinity column, and Western blotting was used to compare the amount of HCN1 in the pool of surface proteins versus total cellular proteins. Co-transfection of GFP-TRIP8b (1a-4) with HCN1_WT_ increased the surface expression of HCN1_WT_ by fivefold. Consistent with the qualitative imaging analysis, HCN1_MUT_ also increased surface expression over HCN1_WT_ but only by twofold (Fig. 6). In conclusion, these data demonstrate that the di-arginine motif functions in the intact channel by limiting the amount of channel allowed to traffic from the ER to the plasma membrane.

## Discussion

The central finding of this study is that HCN1 contains a di-arginine ER retention signal, conserved across species, that influences the amount of channel trafficked to the plasma membrane of both undifferentiated and polarized cells. As one of the carriers of I_h_, HCN1 plays essential roles in neuronal cells by modulating membrane excitability and signal integration thereby influencing a diverse set of processes. Controlling the amount of HCN1 available in the plasma membrane has a direct effect on the amount of current that can be conducted in response to various stimuli.

To date, a number of missense mutations reported in human HCN1 demonstrate that disrupting this channel leads to various forms of epilepsy [46, 47]. Most of these mutations occur near the pore, thus causing altered activity and concomitantly reducing expression of the channel. In places such as the retina, it seems that excess HCN1 is available as the 40 % reduction in HCN1 heterozygous mice or TRIP8b knockout mice does not grossly affect visual function [23]. This implies that an additional insult that further reduces the amount of surface expressed channel or a mutation in the di-arginine motif that would enhance the surface expression of a channel with altered activity could tip the balance and cause even more severe disorders.

The di-arginine ER retention signal is found in unrelated channels and receptors including NMDA receptors, GABA_B_ receptors and ATP-sensitive potassium channels (K_ATP_) [48–52]. It has been proposed that di-arginine motifs are only functional if found within approximately 16–46 Å from the membrane [53]. The di-arginine motif in HCN1 is separated from the membrane by the C-linker and CNBD that together occupy 52 Å in the crystal structure of HCN2 [24]. This does not strike us as significantly different and predict that as more structures for membrane proteins become available, other di-arginine motifs will be found within this range.

The mechanism through which di-arginine motif-containing proteins are retained in or retrieved to the ER is not well understood. We favor the hypothesis that exposure of the di-arginine motif allows for weak or transient interactions with as yet uncharacterized ER proteins and that subunit assembly masks the di-arginine motif, thus allowing escape from the ER. This is the model proposed for the ATP-sensitive potassium (K_ATP_) channels, which require four inward rectifier potassium (K_ir_) channel subunits and four sulphonylurea receptor (SUR) subunits for proper assembly. Both K_ir_ and SUR contain the di-arginine ER retention signal, and neither subunit is expressed at the plasma membrane alone. Assembly into a heterooctamer likely sterically hinders the di-arginine motifs, allowing trafficking to the plasma membrane [48].

Could the di-arginine motif in HCN1 function by a similar mechanism? We observed that fragments of HCN1 containing the di-arginine motif but incapable of forming a channel show robust ER localization. However, in intact channels, a mixture of plasma membrane and ER localized channels was observed with an intact di-arginine motif shifting the balance more toward the ER. This supports the idea that the di-arginine motif in HCN1 could function similar to the K_ATP_ channels. The only known subunit that heterooligomerizes with HCN1 in photoreceptors is TRIP8b, which we have shown is dispensable for surface expression of HCN1 in the retina [23]. Therefore, the di-arginine ER retention signal present in HCN1 is more likely monitoring homooligomerization in photoreceptors rather than heterooligomerization.

Specific HCN channels are enriched in particular brain regions although most show some degree of overlap such as hippocampal neurons, which express both HCN1 and HCN2 [54–58]. It is speculated that in these neurons HCN1 and HCN2 are forming heteromeric channels, which in model expression systems have distinct biophysical properties. Since HCN2 and HCN4 lack a di-arginine motif heterooligomerization with HCN1 would provide an additional level of control to the trafficking of these h-channel heterooligomers. On the other hand, HCN3 contains a putative di-arginine motif (RKR) sequence following the CNBD and interestingly, HCN3 has been shown to localize primarily to ER-like structures when transfected in opossum kidney cells [59]. Clearly, the di-arginine motif in HCN1 regulates trafficking out the endomembrane system, but the role of this motif in the trafficking of related channels remains to be dissected.

An alternative mechanism for the action of the di-arginine motif would be for it to allosterically respond to post-translational modifications signaling maturation of the channel, such as glycosylation or phosphorylation. HCN1 is *N*-glycosylated in the third extracellular loop. This modification is seen as a 15 kDa shift in the mobility of HCN1 in Western blots [54, 56]. As expected, biotinylation assays have shown an increase in the amount of the glycosylated form in the surface pool, from roughly 10 % of the total HCN1 to nearly half. [23]. The amount of unmodified HCN1 at the surface indicates that if glycosylation is a maturation signal it may be needed on only one or two subunits of the intact channel.

Tyrosine phosphorylation of the C-linker in HCN2 and HCN4 by Src kinase regulates channel gating [60, 61], but it is hard to imagine that this effect is functioning at the ER and influencing trafficking of these channels. Notably, the di-arginine motif is adjacent to a conserved tyrosine that is flanked by three to eight serines or tyrosines in an arrangement that in the mammalian, though not the *Xenopus* sequences, matches to the consensus phosphorylation sequences for several abundant kinases [43, 62, 63]. While experimental validation is required, it is tempting to think that phosphorylation at one or more of these sites contribute to the function of the di-arginine motif as in the case of the calcium-sensing receptor and NMDA receptor [51, 64].

Another outstanding question is how regulating trafficking in the early secretory pathway by the di-arginine motif is coordinated with other modulators of HCN1 trafficking. In this study, we focused on the role of the C-terminus exactly because this region has been found to contain the binding sites for proteins proposed to regulate HCN trafficking in various cells [1]. We had previously been surprised to find that loss of TRIP8b did not decrease the surface expression of HCN1 in the retina (beyond that dictated by the reduction in total HCN1 protein available) as it does in cortical neurons [23]. In this study that provided an advantage in that by using photoreceptors as one of our model systems we were able to reveal a previously undiscovered mode of regulation. When we mutated the di-arginine motif in our reporter-based assay, we observed that the reporter filled the outer segments but was not specifically accumulating in the plasma membrane of the inner segment, where the endogenous channel is found. This suggests one of two scenarios: either additional signals operating through the C-terminus of HCN1 can only function in the context of a full-length molecule and/or properly folded channel, or regions of HCN1 outside of the C-terminus carry signals that influence its trafficking. Future studies designed to distinguish among these possibilities would be valuable in mapping out the trafficking pathway of HCN1 from ER to plasma membrane which in turn should pinpoint targets that can be manipulated to modify the amounts of HCN1 that are trafficked to the surface in either healthy or diseased cells.

In summary, we identified a novel di-arginine ER retention signal in the C-terminus of HCN1. The signal functions to limit the amount of HCN1 that exits from the ER to the the plasma membrane. We propose this is part of the tight control imposed on HCN1 trafficking given that altered surface expression of HCN1 is often observed in diseases such as epilepsy [56].

### Electronic supplementary material

Below is the link to the electronic supplementary material.

Supplemental Fig. 1 Primers used to clone reporter: HCN1 constructs *A)* Cartoon of constructs from Fig. 1 with the location and names of cloning primers indicated. The portion of the primer in red corresponds to the 3′ end of the reporter, the portion in black corresponds to the HCN1 fragment. After splicing by overlap extension PCR the products were digested with AgeI and NotI then ligated into the XOP5.5 vector *B)* Sequences of primers (TIFF 324 kb)

Supplemental Fig. 2 Localization of HCN1 relative to the ER in HEK293 cells Location of HA-HCN1_WT_ (A) or HA-HCN1_MUT_ (B) was determined by co-labeling with anti-HA antibodies (green) and the ER marker calnexin (red). Nuclei (blue), and scale bars, 10 μm (TIFF 422 kb)

## Abbreviations

HCN1: Hyperpolarization and cyclic nucleotide-gated channel 1
ER: Endoplasmic reticulum
TRIP8b: Tetratricopeptide repeat-containing Rab8b interacting protein
CNBD: Cyclic nucleotide-binding domain
K_ATP_: ATP-sensitive potassium channels
K_ir_: Inward rectifier potassium channel
SUR: Sulphonylurea receptor
