# Supplementary figures and images for: A di-arginine ER retention signal regulates trafficking of HCN1 channels from the early secretory pathway to the plasma membrane
Source: Cell Mol Life Sci. 2014 Aug 21;72(4):833–43. doi: 10.1007/s00018-014-1705-1 (PMC4309907; doi:10.1007/s00018-014-1705-1)

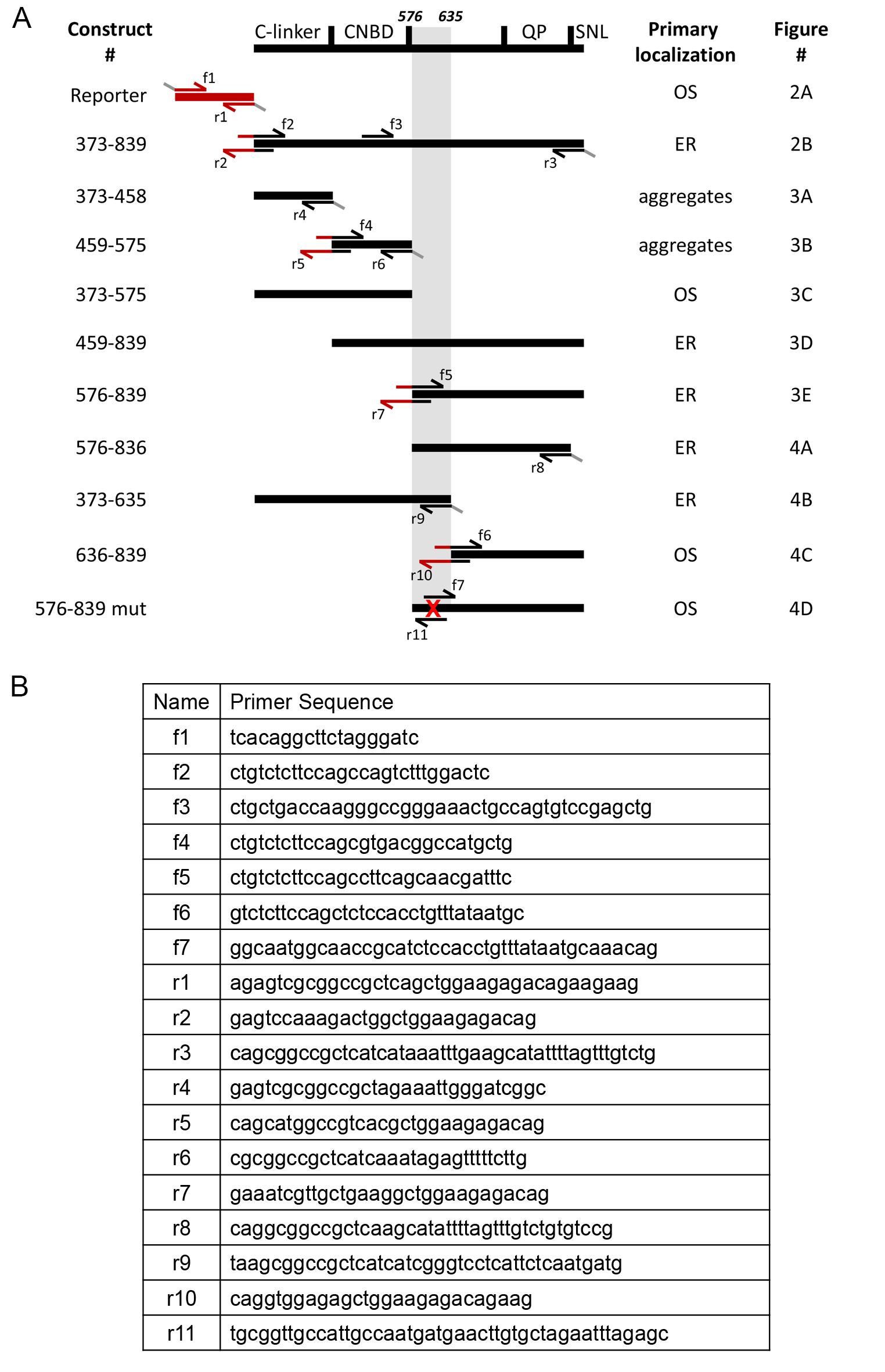

Supplement: Supplementary file 1 — Supplemental Fig. 1 Primers used to clone reporter: HCN1 constructs A) Cartoon of constructs from Fig. 1 with the location and names of cloning primers indicated. The portion of the primer in red corresponds to the 3′ end of the reporter, the portion in black corresponds to the HCN1 fragment. After splicing by overlap extension PCR the products were digested with AgeI and NotI then ligated into the XOP5.5 vector B) Sequences of primers (TIFF 324 kb) [file 18_2014_1705_MOESM1_ESM.tif]

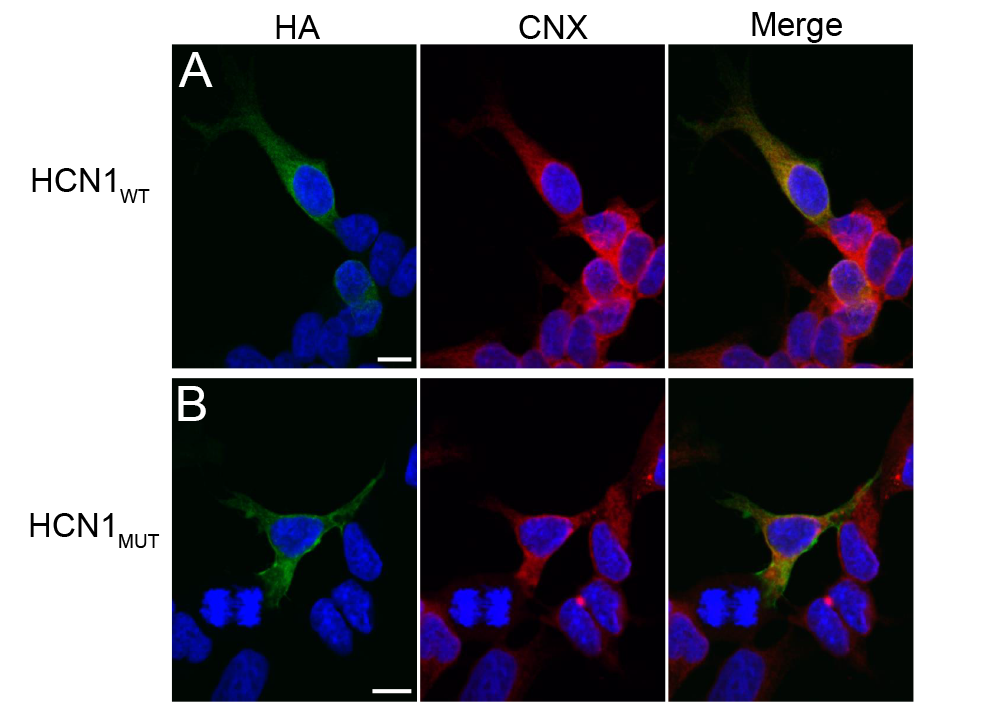

Supplement: Supplementary file 2 — Supplemental Fig. 2 Localization of HCN1 relative to the ER in HEK293 cells Location of HA-HCN1WT (A) or HA-HCN1MUT (B) was determined by co-labeling with anti-HA antibodies (green) and the ER marker calnexin (red). Nuclei (blue), and scale bars, 10 μm (TIFF 422 kb) [file 18_2014_1705_MOESM2_ESM.tif]
